# Association of Mid-Life Changes in Body Size, Body Composition and Obesity Status with the Menopausal Transition

**DOI:** 10.3390/healthcare4030042

**Published:** 2016-07-13

**Authors:** Carrie Karvonen-Gutierrez, Catherine Kim

**Affiliations:** 1 Department of Epidemiology, School of Public Health, University of Michigan, Ann Arbor, MI 48109, USA; 2 Departments of Medicine and Obstetrics & Gynecology, University of Michigan, Ann Arbor, MI 48109, USA; cathkim@umich.edu

**Keywords:** women, menopause, obesity, weight, body composition

## Abstract

The mid-life period is a critical window for increases in body weight and changes in body composition. In this review, we summarize the clinical experience of the menopausal transition by obesity status, and examine the evidence regarding the menopausal transition and reproductive hormones effects on body weight, body composition, or fat distribution. Mid-life obesity is associated with a different menopausal experience including associations with menstrual cycle length prior to the final menstrual period (FMP), age at the FMP, and higher prevalence of vasomotor symptoms. The menopausal transition is associated with weight gain and increased central body fat distribution; the majority of evidence suggests that changes in weight are due to chronological aging whereas changes in body composition and fat distribution are primarily due to ovarian aging. Continuous and regular physical activity during mid-life may be an efficacious strategy to counteract the age-related and menopause-related changes in resting energy expenditure and to prevent weight gain and abdominal adiposity deposition.

## 1. Introduction

Obesity is one of the most pressing threats to public health given its increasing prevalence globally [1] and its significance as a major risk factor for a variety of chronic conditions including diabetes and cardiovascular disease [2,3]. Worldwide, 13% of adults age 18 years and older were obese (body mass index (BMI) ≥ 30 kg/m^2^)) in 2014 [1] and the prevalence of adult obesity in the United States exceeds 34% [4]. Both globally and nationally, women experience higher rates of obesity than men [1,5]. Epidemiologic evidence suggests that among women, the mid-life period is a critical window for increases in body weight and changes in body composition. In this review, we discuss the evidence as well as the potential mechanisms driving the association. First, we review the most recent nomenclature for the characterization of the menopausal transition followed by standard definitions of obesity and measurement of body composition. Next, we describe how the clinical experience of the menopausal transition (i.e., menstrual cycle length, age at the final menstrual period (FMP), and vasomotor symptoms) differs by obesity. Third, we review the studies describing how body composition changes with menopausal status. Fourth, we discuss the biologic underpinnings of these associations including advancing chronologic and ovarian age; changes in sex steroids and other sex hormones; and the possibility that obesity precedes changes in sex hormone profiles. We conclude with suggestions for clinical management and future research.

## 2. Nomenclature of the Menopause, Obesity, and Body Composition

The hallmark of menopause is the FMP, defined as the last menstrual period followed by 12 months of amenorrhea. However, the menopausal transition begins 5–10 years before a woman’s FMP and is characterized by menstrual cycle variability and fluctuations in reproductive hormone levels [6]. The transition ends years after the FMP and is characterized by stabilization in reproductive hormone levels. The 2011 International Stages of Reproductive Aging Workshop (STRAW+10) has incorporated findings from longitudinal studies of hormone data and bleeding characteristics to develop a framework for staging menopause that has been widely implemented in contemporary clinical and research settings [6]. Prior to the adoption of STRAW+10, the stages of menopause were commonly based upon bleeding patterns [7]. Postmenopause was the period of time following the FMP. Perimenopause preceded the FMP and was characterized by menstrual cycle irregularity. Premenopause preceded perimenopause and was characterized by regular menses. While the STRAW classification system still relies upon the menstrual cycle to define stages of menopause, the modified staging recognizes that particular changes in menstrual flow can define late reproductive years versus early reproductive years and early versus late menopausal transition stages. In addition, early versus late postmenopausal transitions can be defined using sex steroids and other hormone markers.

More specifically, the late reproductive stage is characterized by subtle changes in flow length as compared to previously regular cycles, the early menopausal transition is distinguished as persistent 7 day differences in the length of consecutive cycles, and the late menopausal transition is characterized as intervals of amenorrhea greater than 60 days. In the early postmenopause, sex hormones are variable but then stabilize several years after the FMP. Data from two longitudinal studies of mid-life women, the Michigan Bone Health and Metabolism Study (MBHMS) and the Study of Women’s Health Across the Nation (SWAN) have documented that levels of estradiol (E2) are fairly stable up until 2 years prior to the FMP, followed by a 4-year sharp decline spanning until 2 years after the FMP [8,9]. Levels of follicle-stimulating hormone (FSH) begin to rise up to 10 years prior to the FMP [8] and rise more quickly approximately 2 years prior to the FMP before stabilizing 2 years after the FMP [9]. Although FSH is accepted as an earlier biomarker of endocrine change during the menopausal transition, its month-to-month variability in the year preceding and following the FMP makes it an imprecise measure of the menopausal transition [8]. As exogenous estrogen and gynecologic surgeries can alter bleeding patterns, women who use hormonal contraception or who have undergone hysterectomy and/or bilateral oophorectomy cannot be classified according to these stages.

Classifications of overweight and obesity status are based upon BMI which is calculated as weight (in kilograms) divided by height squared (in meters). Overweight is defined as BMI between 25.0–29.9 kg/m^2^ and obesity is defined as BMI ≥ 30 kg/m^2^ [1]. While BMI is often used in both research and clinical settings, it is an overall summary measure of body size and thus does not completely capture measures of fat distribution or body composition which have independent predictive power for mortality [10,11,12]. Fat distribution can be assessed crudely using waist circumference, hip circumference and the ratio of waist-to-hip circumference [13], although the accuracy and precision of anthropometry is low compared to imaging [14]. More sophisticated methods for measurement of fat distribution include DEXA (dual energy X-ray absorptiometry), computed tomography (CT) and magnetic resonance imaging (MRI) which can provide precise measures for subcutaneous adipose tissue (SAT) and visceral adipose tissue (VAT) by body region [15]. Ascertainment of fat and lean mass can be quantified using a variety of measures including bioelectric impedance analysis (BIA), DEXA or CT [15]. No standard definitions exist as to what cut points define greater risk for chronic disease or mortality in women.

## 3. Clinical Experience of the Menopausal Transition by Obesity

Mid-life obesity is associated with a different menopausal experience including associations with menstrual cycle length prior to the FMP [16,17], age at the FMP [18,19,20,21,22,23,24], and higher prevalence of vasomotor symptoms [25,26,27,28,29,30,31,32,33,34,35,36]. Women with a higher BMI may experience a longer menopausal transition than those with a lower BMI [37].

The association between higher BMI and longer menstrual cycle length has been noted during the reproductive years [38,39,40,41,42]. This association continues through the menopausal transition. Using SWAN data, Paramsothy et al. have noted that women who were obese had menstrual cycles that were 1.45–4.94 days longer as compared to normal weight women [16]. These results confirm an earlier analysis from the cross-sectional SWAN Daily Hormone Study which included daily first morning void urine samples through one menstrual cycle and found that overweight and obese women had a greater proportion of long cycles (i.e., greater than 33 days) as well as longer follicular phases and shorter luteal phases as compared to normal weight women [17]. In addition, a recent report from the Hungarian Menopause Study found that the irregular bleeding as well as prolonged bleeding during the menopausal transition was more common among women with higher amounts of body fat and obesity as compared to non-obese women [43].

Whether obesity impacts the timing of the FMP is controversial. The majority, but not all [44,45,46] of cross-sectional studies suggest that higher BMI [18,19,20,21,22,23,24], weight [24,47,48], waist-to-hip ratio [49], or skinfold thickness [50] is associated with later age at the FMP. A meta-analysis of self-reported BMI and age at natural menopause using data from 9 studies found that obese women were 15% (hazard ratio (HR) = 0.85, 95% CI 0.81, 0.90) less likely to become postmenopausal at a given age as compared to women with a normal BMI after controlling for smoking [51]. As the FMP is defined after one year of amenorrhea, these studies rely on participant recall of body mass at the time of the FMP. Some cross-sectional studies have tried to address these issues by asking about premenopausal body weight and have confirmed the association of obesity and later age at menopause. In the United Kingdom-based Breakthrough Generations Study conducted from 2003–2011 among more than 50,000 women age 40–98 years of age, self-reported weight at age 40 and weight gain between ages 20–40 years was associated with a later age at menopause [52]. Similarly, in the Shanghai Women’s Health Study among more than 30,000 women age 40–70 years, self-reported BMI at age 20 predicted later age at menopause [53]. In contrast, most longitudinal studies have found no association [54,55,56,57] or at best, a very modest association [18,58,59] between obesity and age at FMP.

The contrast between the cross-sectional studies and the longitudinal studies may be due, in part, to issues with recall of BMI at the age of FMP as well as confounding by factors such as smoking [51]. Another possible explanation for the conflicting results is the inability to define age at natural menopause among women with surgical menopause (bilateral oophorectomy) or hysterectomy. Obesity is associated with increased likelihood of having a surgical menopause [44] and increased weight gain is a common complaint among women following hysterectomy [60]. Thus, failure to account for risk of surgical menopause or hysterectomy may also contribute to inaccurate estimates of the age at FMP for a particular study population.

Obese women have a more symptomatic menopausal transition as compared to normal weight women [61]. In the large SWAN cross-sectional study among more than 16,000 women age 40–55 years, BMI was positively correlated with self-reported hot flashes or night sweats, urine leakage, and joint stiffness or soreness [62]. In the Women’s Health Initiative, urogenital symptoms including vaginal discharge, itching, and irritation were more 2–4 times more common among obese women as compared to normal weight women [63]. Perhaps the most commonly studied menopausal symptom associated with obesity is vasomotor symptoms (VMS). Although obesity was once thought to be protective for VMS given more blunted changes in E2 during the menopausal transition among obese women [9,64], it is now well-appreciated that obesity is associated with greater VMS prevalence during the mid-life [25,26,27,28,29,30,31,32,33,34,35,36] although studies do not support an association of BMI and VMS among post-menopausal women [27,65,66,67]. The mechanism for this relationship is not well understood but the prevailing hypothesis is that adipose tissue itself has an insulating effect which may inhibit heat dissipation [68,69]. In fact, findings from longitudinal studies demonstrate that gains in body fat over time predict risk [70,71] and frequency of VMS [72].

## 4. Menopausal Status and Body Composition

The menopausal transition is associated with changes in other aspects of body composition aside from increasing BMI. First, weight gain is a common complaint among mid-life women [73,74,75], particularly during the menopausal transition [76,77,78,79,80,81,82,83,84,85]. Second, women have relatively greater SAT prior to the FMP versus relatively greater VAT deposition after the FMP [86,87], which reflects increased central body fat distribution [80,85,88,89,90,91,92,93]. During the menopausal transition, the visceral fat depot increases from being 5%–8% of total body fat during the premenopausal period to being 15%–20% of total fat during the postmenopausal period [94,95]. Finally, the menopausal transition is associated with decreased lean muscle mass [80,92,96,97,98].

Whether these increases are due to advancing age or alterations in reproductive hormones has been debated [99]. While there is not a consensus opinion, the majority of evidence supports changes in BMI and weight with aging and changes in body composition with reproductive hormone changes. Multiple reports note that women tend to gain weight with age, regardless of the menopausal transition [43,100,101,102]. Among women in SWAN age 40–55 years, the average 3-year increase in body weight was 4.5 pounds [102]; similarly, the Healthy Women’s Study reported that women gain 0.7 kg/year (1.5 pounds/year) during their 5th and 6th decades of life [78]. Other reports have noted that body size and distribution measures changed over the menopause but were largely explained by age [54,77,103].

In contrast, multiple reports suggest that midlife changes in body composition (as opposed to BMI or weight) are likely not entirely due to age. Studies that use more sensitive measures such as DEXA [76,88,89,90,97,98,104,105,106,107,108] or CT or MRI [87,109,110,111] have observed an increase in abdominal tissue redistribution during the postmenopause. Menopausal increases in VAT independent of age have been confirmed in cross-sectional [112,113,114,115] and longitudinal studies [89,92,93,107,116]. For example, in a study of 23 women who were assessed while in the premenopause and the postmenopause (8 years apart) [117], within individual women there was no change in weight or waist circumference from the pre- to postmenopausal period after adjusting for age but there were statistically significant increases in total abdominal fat, SAT and VAT [117]. In a larger study from the Michigan site of SWAN, Sowers et al. [80] demonstrated a 6% increase in waist circumference, a 10% increase in fat mass and a 1% decrease in skeletal muscle mass over a 6-year period around the FMP [80] that persisted after adjustment for chronologic age.

## 5. Mechanisms Linking Body Composition and Menopausal Status

Since the menopausal transition is characterized by changing reproductive hormone levels, sex hormones are obvious candidates for changing body composition. The impact of sex hormones upon body composition has been demonstrated in both animal models as well as women undergoing surgical menopause. In animal experiments, oophorectomized rats had increased obesity [118,119], potentially due to increased food intake [120,121], decreases in adipose tissue lipolysis [122], decreased physical activity [123] and reduced energy expenditure [121,124]. Treatment with estrogen attenuates the ovariectomy-induced weight gain and abdominal adiposity deposition [125,126]. Among humans, the abrupt changes in E2 and androgen production following surgical menopause may increase the risk for weight gain [127,128]. In data from SWAN, women with a surgical menopause had 5 times increased odds (odds ratio (OR) = 5.07, 95% CI 2.29, 11.20)) of developing severe obesity as compared to women who remained premenopausal [82]. Further, also in SWAN, and the rate of change in BMI increased more rapidly among women with surgical menopause as compared to women with natural menopause [84].

However, the interaction between sex hormones and obesity during the menopausal transition is complex, since adipose tissue is active metabolically and associated with lower sex hormone-binding globulin (SHBG) levels [61,96,112,129,130]. Thus, adipose tissue affects reproductive hormone levels even as reproductive hormone levels may affect adipose tissue deposition [115,131,132].

During the premenopausal period, obese women have lower levels of E2 and FSH compared to non-obese women [64,129,133,134,135]. While the overall pattern and timing of changes in E2 and FSH are not affected by obesity status [9], both SWAN and the Penn Ovarian Aging Study (POAS) have demonstrated that the characteristic drop in E2 levels during the menopausal transition is more rapid among non-obese women as compared to obese women [64,136]. The more blunted rate of change in E2 during the menopausal transition among obese women as compared to non-obese women reflects observed lower premenopausal E2 levels but higher postmenopausal E2 levels among obese women [9,64]. Observations of lower E2 levels among premenopausal obese women and higher E2 levels among postmenopausal obese women have been confirmed in several studies including the Women’s Ischemia Syndrome Evaluation (WISE) study [137], a cohort comprised of women from the New York Women’s Health Study, the Northern Sweden Health and Disease Study and the Study of Hormones and Diet in the Etiology of Breast Cancer [138] and women from the Philadelphia area [139]. The biological underpinnings of higher E2 among obese postmenopausal women may reflect the shift to adipose tissue as the primary source of E2 after the FMP; with the cessation of ovarian function, most postmenopausal E2 is from the aromatization of androgens within the adipose tissue [140]. Given that obese women have larger adipose tissue depots, they are able to produce larger amounts of E2 [140].

Less is known however about the cause for lower E2 and inhibin B levels among premenopausal obese women. Several potential reasons have been identified including adiposity-induced suppression of sex hormone binding globulin synthesis [141,142,143] which led to greater clearance of E2 and a possible negative effect of obesity on granulosa cell function thereby decreasing inhibin B levels.

Obesity is associated with changes in other reproductive hormones during the menopausal transition. Prior to the FMP, obese women have higher levels of testosterone than non-obese women [129]. Longitudinal studies suggest that changes in androgens [82,116,144,145] predict obesity risk. During the first 9 years of follow-up in SWAN, increases in free androgen index (FAI) as well as decreases in SHBG predicted incident obesity status [82] independent of age. Associations of bioavailable androgens and adiposity have been confirmed in the Chicago SWAN Fat Patterning Study which included over 240 women with 4 years of follow-up. Higher increases in FAI were associated with larger increases in VAT [144].

Prior to the FMP, obese women also have lower levels of luteinizing hormone (LH), inhibin B [64,134,146], estrone, progesterone [129] and anti-Müllerian hormone levels [147]. As women transition through menopause, the relationships between obesity and sex hormones change. Among more than 400 Black and White women from the longitudinal POAS who were followed for 12 years, obese women had lower levels of inhibin B during the pre-menopausal period but higher inhibin B levels during the postmenopausal period as compared to non-obese women [64]. Although mechanisms are speculative, it has been hypothesized that adiposity-induced suppression of SHBG synthesis leads to greater clearance of E2 and a possible negative effect of obesity on granulosa cell function, which in turn decreases inhibin B levels [141,142,143].

Other reports underlie further the importance of changing body composition upon the menopausal hormonal milieu. In a landmark paper using data from more than 1500 women from SWAN, Wildman et al. [148] demonstrated that current waist circumference predicted future levels of SHBG, testosterone and FSH but that changes in these hormones did not impact future waist circumference [148]. They also noted that current waist circumference predicted future E2 [148]. Thus, this analysis suggests that changes in adiposity precede changes in sex steroids during the menopausal transition.

If not solely ovarian sex steroids, what factors cause a shift in fat distribution with change in the menopausal status? One candidate hormone is FSH. Due to the loss of negative feedback from inhibin, serum FSH levels increase from pre- to postmenopause. While FSH receptors were originally thought to be restricted to the gonads, FSH receptor is present in visceral fat in both men and women [149]. In vitro administration of FSH to preadipocytes in mice resulted in redistribution of visceral fat mass and an increase in adipocyte lipid droplets and adipocyte lipid synthesis [149]. Moreover, serum levels of adipokines (cytokines secreted by adipose tissue) including leptin and adiponectin and lipid subgroups including triglycerides were altered, thereby suggesting that FSH might stimulate fat distribution and contribute to a pro-inflammatory milieu [149]. Another candidate hormone is cortisol, which increases visceral fat expansion [150]. Although serum levels of cortisol are not different in postmenopausal women compared to premenopausal women, serum estrogen levels may modify the expression of cortisol receptors in visceral fat and local levels of glucocorticoids.

Obesity could also contribute to the experience of the menopausal directly through adipokines including leptin, adiponectin, and resistin given documented differences in these adipokines by menopausal stage [151]. A SWAN substudy found that such adipokines were associated with VMS, particularly early in the menopausal transition [31]. Other prospective studies have also found that serum levels of C-reactive protein, tissue plasminogen activator, leptin, and adiponectin change from before to after the final menstrual period, with adverse inflammatory profile more common after compared to before the menopause and with strong correlations with visceral adiposity [93]. However, it was unclear whether inflammatory markers resulted in changes in the timing of the final menstrual period or other sex steroids or vice versa.

## 6. Implications for Therapy

Exogenous estrogen therapy has been suggested as a potential therapy to blunt weight gain and unfavorable body composition changes. Since VAT is the more at-risk fat distribution phenotype, exogenous hormone therapy has been postulated to be beneficial for the maintenance of a healthy body typology during the mid-life. Several studies have demonstrated a beneficial effect of hormone therapy in the reduction of central adiposity [108,152,153,154,155,156,157]. A meta-analysis of more than 100 randomized controlled trials among menopausal women without diabetes concluded that oral and transdermal estrogen was associated with reductions in abdominal fat [158]. However, the increased risk of stroke and breast cancer with oral estrogen replacement in postmenopausal women has limited the use of oral estrogen for chronic disease prevention [159]. Thus, estrogen therapy may alter visceral fat distribution. However, such therapy adversely affects outcomes, and thus the benefits of therapy are outweighed by the other changes induced by estrogen therapy. These include, but are not limited to, increased thrombosis and inflammation. At this point in time, the biomarkers that might mediate the increased risk of hormone therapy are still largely unknown [160].

The use of transdermal estradiol in younger women has been the subject of much interest. Randomized trials have not demonstrated increased risk of subclinical atherosclerosis with randomization to transdermal formulations [161] but data on incident malignancy or cardiovascular disease events is not yet available. Thus, transdermal estrogen therapy in the perimenopause has been used primarily for relief of VMS until additional data are available [162].

Due to their low risk, the mainstays of obesity prevention include calorie restriction and physical activity. Findings from genetic studies show low heritability for waist circumference and waist-to-hip ratio among premenopausal women, suggesting that most of the phenotypic variation during this time is due to environmental causes [163]. Sedentariness predicts weight gain and abdominal obesity risk over and above the effects of aging and menopause [164] and longitudinal decreases in total energy expenditure during the mid-life have been primarily attributed to decreases in physical activity [165]. Age-related declines in resting energy expenditure are not observed among those women that exercise regularly [89]. Thus, continuous physical activity during the mid-life may be an efficacious strategy to counteract the age-related [166] and menopause-related decreases [89] in resting energy expenditure and prevent weight gain and abdominal adiposity deposition. In fact, a recently published meta-analysis of 8 walking intervention studies among peri- and postmenopausal women demonstrated statistically significant improvements in BMI, body weight, and body fat percentage as compared to no-exercise groups [167]. Further, recent work by Riou et al. found that the quantity (i.e., amount of time spent) rather than the intensity of physical activity may be most efficacious strategy promoting lower adiposity and maintenance of body composition over the menopausal transition [168]. Although little data exists as to whether dietary modification can affect fat distribution during the menopausal transition, evidence suggests that exercise interventions as compared to dietary interventions may be superior for the promotion of a healthy body composition profile including preservation of lean mass and greater decreases in body fat [169].

## 7. Conclusions

Due to the high prevalence of obesity in midlife, most women are overweight as they transition through the menopause. Examinations of the interactions between adiposity and sex hormone changes have expanded our understanding of the endocrinologic activity of adipose tissue as well as the contribution of sex steroids to body composition in women. The complex interplay between body composition changes and sex hormones in milieu suggests that multiple approaches to healthy body composition may be useful. Further investigation of pre- and perimenopausal management with estrogen therapy is also needed to understand how this affects the menopausal transition and future symptoms. Although prevention of weight gain through behavioral changes is the mainstay of therapy, further investigation of the efficacy and long-term safety of estradiol formulations is needed.

## Abbreviations

The following abbreviations are used in this manuscript:

FMP: Final Menstrual Period
BMI: Body Mass Index
STRAW+10: 2011 International Stages of Reproductive Aging Workshop
MBHMS: Michigan Bone Health and Metabolism Study
SWAN: Study of Women’s Health Across the Nation
E2: Estradiol
FSH: Follicle-Stimulating Hormone
CT: Computed Tomography
MRI: Magnetic Resonance Imaging
SAT: Subcutaneous Adipose Tissue
VAT: Visceral Adipose Tissue
BIA: Bioelectric Impedance Analysis
DEXA: Dual Energy X-ray Absorptiometry
HR: Hazard Ratio
VMS: Vasomotor Symptoms
OR: Odds Ratio
SHBG: Sex Hormong-Binding Globulin
POAS: Penn Ovarian Aging Study
WISE: Women’s Ischemia Syndrome Evaluation study
